# *WUSCHEL-RELATED HOMEOBOX4* acts as a key regulator in early leaf development in rice

**DOI:** 10.1371/journal.pgen.1007365

**Published:** 2018-04-23

**Authors:** Yukiko Yasui, Yoshihiro Ohmori, Yumiko Takebayashi, Hitoshi Sakakibara, Hiro-Yuki Hirano

**Affiliations:** 1 Department of Biological Sciences, School of Science, The University of Tokyo, Bunkyo-ku, Tokyo, Japan; 2 RIKEN Center for Sustainable Resource Science, Suehiro, Tsurumi, Yokohama, Japan; 3 Graduate School of Bioagricultural Sciences, Nagoya University, Chikusa, Nagoya, Japan; University of California Berkeley, UNITED STATES

## Abstract

Rice (*Oryza sativa*) has long and narrow leaves with parallel veins, similar to other grasses. Relative to *Arabidopsis thaliana* which has oval-shaped leaves, our understanding of the mechanism of leaf development is insufficient in grasses. In this study, we show that *OsWOX4*, a member of the *WUSCHEL-RELATED HOMEOBOX* gene family, plays important roles in early leaf development in rice. Inducible downregulation of *OsWOX4* resulted in severe defects in leaf development, such as an arrest of vascular differentiation, a partial defect in the early cell proliferation required for midrib formation, and a failure to maintain cellular activity in general parenchyma cells. In situ analysis showed that knockdown of *OsWOX4* reduced the expression of two *LONELY GUY* genes, which function in the synthesis of active cytokinin, in developing vascular bundles. Consistent with this, cytokinin levels were downregulated by *OsWOX4* knockdown. Transcriptome analysis further showed that *OsWOX4* regulates multiple genes, including those responsible for cell cycle progression and hormone action, consistent with the effects of *OsWOX4* downregulation on leaf phenotypes. Collectively, these results suggest that *OsWOX4* acts as a key regulator at an early stage of leaf development. Our previous work revealed that *OsWOX4* is involved in the maintenance of shoot apical meristem in rice, whereas *AtWOX4* is specifically associated with the maintenance of vascular stem cells in *Arabidopsis*. Thus, the function of the two orthologous genes seems to be diversified between rice and *Arabidopsis*.

## Author summary

Leaves are major photosynthetic organs in plants, and their sizes and shapes are diverse in angiosperms. Proper leaf development is crucial not only for plant body plan but also for efficient photosynthesis. Similar to other grasses, rice has long and narrow leaves with parallel veins, which are distinct from *Arabidopsis* leaves. Our understanding of the mechanism underlying leaf development in rice, in particular cell proliferation and differentiation in early development, is insufficient relative to *Arabidopsis*. Members of the *WUSCHEL-RELATED HOMEOBOX* (*WOX*) gene family play essential roles in plant development, such as stem cell maintenance in the shoot and root apical meristems and regulation of the leaf margin specification. In this study, we show that *OsWOX4* regulates not only tissue differentiation but also cellular activity in early leaf development. *OsWOX4* seems to promote vascular differentiation through the action of a plant hormone, cytokinin, and cell proliferation responsible for midrib formation. These results suggest that *OsWOX4* acts as a key regulator at an early stage of leaf development.

## Introduction

Proper leaf development in plants is crucial not only for their body plan but also for efficient photosynthesis. Plants are sessile organisms that evolve morphological leaf traits by optimization to their respective natural habitats. Leaves are initiated at the flank of the shoot apical meristem (SAM), which harbors a group of stem cells at its apical region [1]. Next, leaf primordia start growing out via cell proliferation, and differentiate into several types of tissue [2]. Leaves are diverse in their shapes and venation patterns in angiosperms. The molecular mechanism of leaf development has been well studied in *Arabidopsis thaliana*, which has oval-shaped leaves with reticulated veins [3]. By contrast, our understanding of leaf development in monocots such as grasses, which have long and narrow leaves with parallel veins, remains limited despite their agronomic importance. In particular, there is less information about the key genes that regulate early leaf development including cell proliferation and tissue differentiation in leaf primordia.

*WUSCHEL-RELATED HOMEOBOX* (*WOX*) genes, which encode plant-specific transcription factors, have important functions in various developmental processes, such as stem cell maintenance, embryogenesis and leaf development [4–9]. In *Arabidopsis* leaf development, *WOX3* and *WOX1* are required for margin development and lateral outgrowth of the leaf blade [10, 11], whereas *WOX4* is involved in maintaining vascular stem cells [12]. In grasses, rice *NARROW LEAF2* (*NAL2*) and *NAL3* and maize *NARROW SHEATH1* (*NS1*) and *NS2*, which encode proteins closely related to *Arabidopsis* WOX3, are involved in leaf margin development, because *nal2 nal3* and *ns1 ns2* double mutants result in narrow leaf phenotypes [13–15]. Thus, genes in the WOX3 clade seem to be functionally conserved in eudicots and monocots. However, functional diversification of other *WOX* genes has been observed. For example, *WUSCHEL* (*WUS*) plays a crucial role in stem cell maintenance in *Arabidopsis* [16–18], whereas its rice ortholog *TILLERS ABSENT1* (*TAB1*) does not have this function but instead is required for initiation of the axillary meristem [19]. In rice, meristem maintenance is regulated by *OsWOX4*, the ortholog of *Arabidopsis WOX4* (*AtWOX4*) [20]. Thus, clarification of the function of the respective *WOX* genes is essential to elucidate the developmental mechanism of each species, in addition to understanding the functional diversification of the *WOX* genes in angiosperm evolution.

In rice, large and small vascular bundles run in parallel in the lateral region of the leaf blade and leaf sheath. These two types of vascular bundle differ in both size and in their tissue organization; for example, a specific tissue called the “mestome sheath” is differentiated in the large bundle [21, 22]. As compared with small vascular bundles, large vascular bundles initiate earlier in leaf primordia, suggesting that the mechanism regulating the timing of initiation differs for the two types of bundle. A few genes, such as *OsHOX1* and *OsPINHEAD/OsZWILLE* (*OsPNH1/OsZLL*), are reportedly expressed in developing vascular bundles [23, 24], although the precise functions of these genes in vascular development have not been elucidated.

The central region of the leaf blade forms a strong structure called the midrib to keep the leaf upright for efficient photosynthesis. The midrib consists of adaxial–abaxial tissues and septum tissues, which surround a few locules. The large and small vascular bundles also run through the midrib region. Formation of the midrib is regulated by *DROOPING LEAF* (*DL*), which encodes a YABBY transcription factor [25]. *DL* promotes cell proliferation in the central region of the leaf primordia to generate enough cells for differentiation into several tissues in the midrib [25, 26]. The function of the *DL*-like *YABBY* genes that regulate midrib formation is conserved in grasses [27, 28]. Because phenotypes such as narrow leaf and rolled leaf are conspicuous, several genes regulating these characteristics have been identified and their functions have been partially characterized [29–31]. Thus, our understanding of the genes that regulate overall leaf morphology such as leaf erectness and shape has been gradually increasing in rice. However, the genes that regulate early leaf development, including cell proliferation in the leaf primordia and early vascular differentiation, remain largely elusive.

The molecular mechanisms underlying vascular development have been well documented in *Arabidopsis* [32, 33]. In brief, polar auxin transport is a key process in the initiation of vascular differentiation [34, 35]. The response to auxin is mediated by the transcription factor MONOPTEROS (MP)/AUXIN RESPONSE FACTOR5 (ARF5), and loss of function of *MP* leads to severe defects in vascular development [36]. MP directly regulates *TARGET OF MONOPTEROS5* (*TMO5*) expression [37], and TMO5 upregulates *LONELY GUY3* (*LOG3*) and *LOG4* genes by forming a heterodimer with LONESOME HIGHWAY [38–40]. The *LOG* genes encode enzymes that activate the phytohormone cytokinin [41, 42], which in turn promotes vascular cell proliferation and patterning in early vascular development [39, 40]. Vascular stem cells in the procambium and cambium are the source of xylem and phloem differentiation [43], and these cells are maintained by *AtWOX4*, because procambial cell proliferation is partially suppressed in the *Arabidopsis wox4* mutant [7, 12].

Our previous study revealed that *OsWOX4* regulates SAM maintenance [20]. However, *OsWOX4* was found to be expressed in the leaf primordia, in addition to the SAM, suggesting that it also has a role in leaf development. In this paper, we examined the effect of *OsWOX4* downregulation specifically on leaf development by inducing RNA silencing after leaf initiation to exclude its effects on SAM function. As a result, we found that pulse downregulation of *OsWOX4* caused defects in vascular differentiation and in early cell proliferation for midrib formation. In addition, *OsWOX4* knockdown repressed the expression of *LOG*-like genes in the developing vasculature and resulted in a reduction of cytokinin content. Transcriptome analysis further showed that *OsWOX4* regulates a number of genes, including those related to cell cycle progression and cellular processes. Consistent with this, cells were abnormally vacuolated in early leaf primordia after longer downregulation of *OsWOX4* and seemed to lose normal cellular activity. Thus, our results demonstrate that *OsWOX4* plays crucial roles in tissue differentiation and cellular activity in early leaf development in rice.

## Results

### Pulse downregulation of *OsWOX4* affects leaf growth

**Fig 1:**
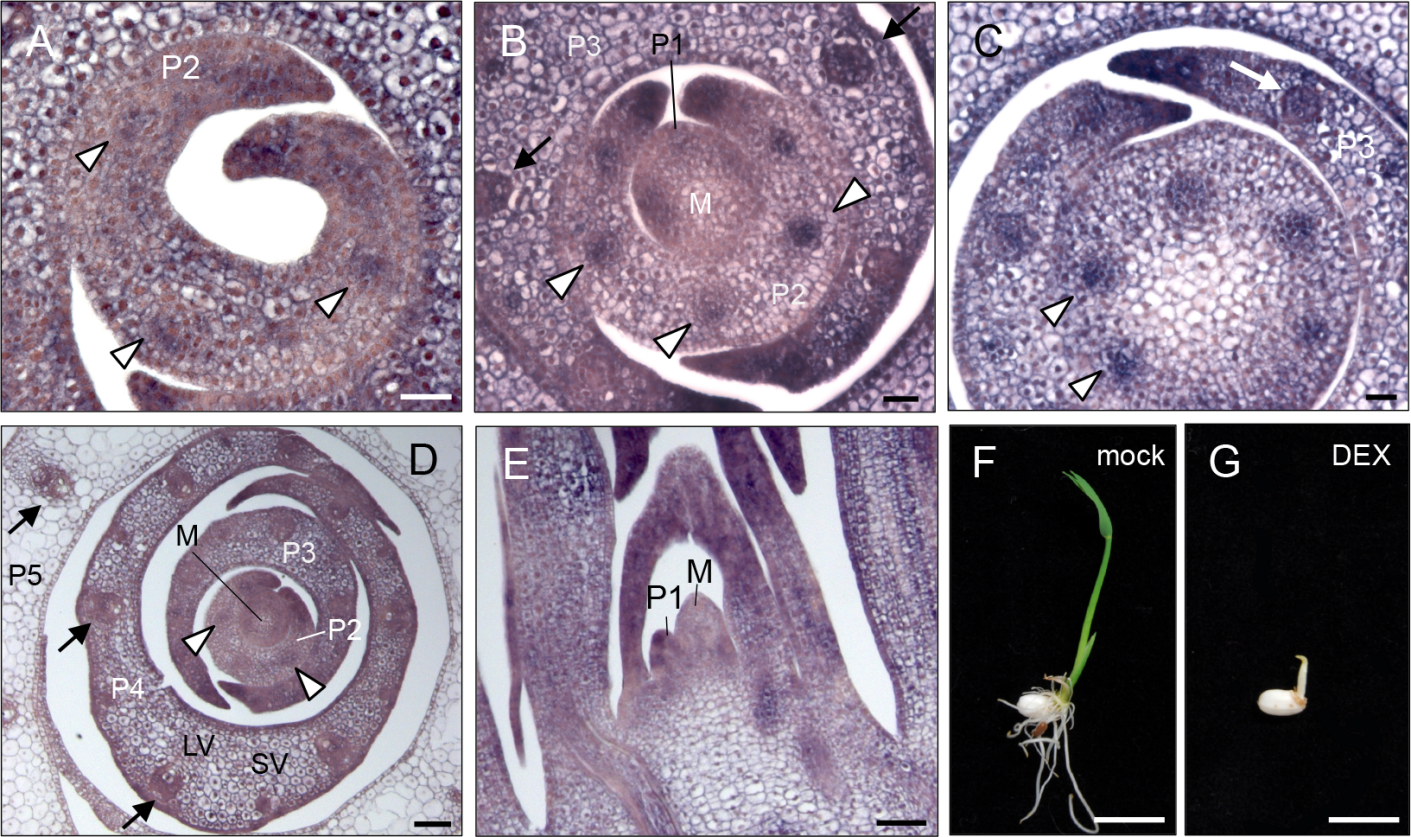
Spatial expression pattern of *OsWOX4* detected by in situ hybridization and the effect of continuous downregulation of *OsWOX4* on leaf growth. (A) to (E) Spatial expression pattern of *OsWOX4* in the wild-type shoot apex. Cross-sections (A to D) and a longitudinal section (E). Cross-section A and C lies above and below the section containing the meristem (B), respectively. Arrowheads indicate the region where differentiation of large vascular bundles should initiate, and arrows indicate developing large vascular bundles. M, SAM; LV, large vascular bundle; SV, small vascular bundle. (F) and (G) Phenotype of the mock- (F) and DEX- (G) treated seedlings. Transgenic plants carrying *pACT1-GVG>OsWOX4*:*RNAi* were treated with DEX for 5 days from germination. Bars = 20 μm in (A) to (C); 50 μm in (D) and (E); 1 cm in (F) and (G).

We first examined the spatial expression pattern of *OsWOX4* in the shoot apex by in situ hybridization. *OsWOX4* was expressed in the leaf primordia, in addition to the SAM (Fig 1A to 1E). *OsWOX4* signals were detected in the region where differentiation of vascular bundles should initiate in the P2 primordium (Fig 1A and 1B). *OsWOX4* was expressed in the developing vascular bundles in P3 and P4 and in putative future vascular bundles just below the SAM (Fig 1C and 1D). In addition to the vasculatures, relatively strong signals were also detected in the margin of leaf primordia (P2-P4) (Fig 1A to 1D). Weak *OsWOX4* expression was also observed in parenchyma cells in P1-P4 (Fig 1A to 1E). In P5, by contrast, *OsWOX4* signals disappeared from the parenchyma cells, although weak expression was observed in the vascular bundles (Fig 1D).

Constitutive downregulation of *OsWOX4* is known to cause premature termination of the meristem [20]. To address the functions of *OsWOX4* in leaf development, therefore, we used an inducible knockdown system in which *OsWOX4* expression was silenced by using a *pACT1-GVG>OsWOX4*:*RNAi* construct induced by dexamethasone (DEX) (S1 Fig) [20]. When DEX was initially applied for 5 days from germination, plant growth was profoundly inhibited such that no leaves were expanded in any transgenic line carrying the *pACT1-GVG>OsWOX4*:*RNAi* construct (Fig 1F and 1G). By contrast, wild type rice showed no abnormality, indicating that DEX treatment alone had no effect on rice growth or leaf development (S2A and S2B Fig).

**Fig 2:**
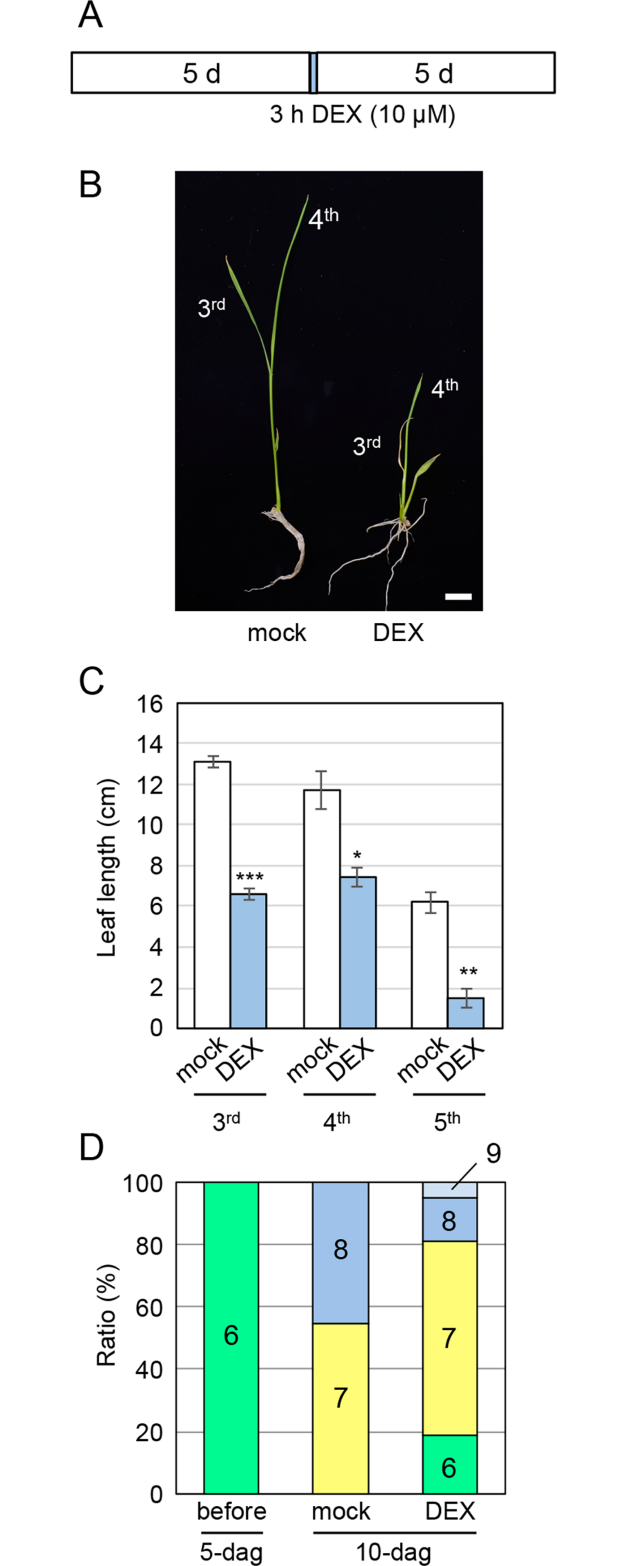
Effects of pulse downregulation of *OsWOX4* on leaf growth. (A) Schematic representation showing the DEX pulse treatment of transgenic plants carrying *pACT1-GVG>OsWOX4*:*RNAi* for *OsWOX4* knockdown. (B) Phenotype of seedlings 5 days after DEX treatment. Bar = 1 cm. (C) Length of the 3^rd^, 4^th^, and 5^th^ leaves (total length of leaf blade and leaf sheath) of DEX- and mock-treated plants. Data are the mean ± SE (*n* = 16 [3^rd^ and 4^th^, mock]; *n* = 17 [3^rd^ and 4^th^, DEX]; *n* = 9 [5^th^, mock]; *n* = 10 [5^th^, DEX]). Student’s t-test, *P < 10^−3^, **P < 10^−5^, ***P < 10^−16^. (D) Percentage of plants producing the indicated number of leaves (including leaf primordia). *n* = 6 (before); *n* = 22 (mock); *n* = 21 (DEX).

To examine the function of *OsWOX4* in leaf development, we established an experimental protocol in which only a pulse of *OsWOX4* downregulation was applied and subsequent plant growth was measured. In this experiment, plants 5 days after germination (5 dag) were treated with DEX for 3 h, and then grown under normal conditions without DEX for a further 5 days (Fig 2A). Leaf phenotypes and histological characteristics were then examined in the 10-dag plants. Leaf growth was clearly inhibited by this pulse downregulation of *OsWOX4* (Fig 2B). We measured the length of the 3^rd^, 4^th^ and 5^th^ leaves in 10-dag plants, which were initiated in the 5-dag plant before DEX treatment (S3A and S3C Fig). The lengths of these leaves at 10 dag were significantly shorter in DEX-treated plants than in mock-treated plants (Fig 2C).

Next, we counted the number of leaves including leaf primordia. We made cross-sections of the seedlings because younger leaves are enclosed inside the older ones in rice. The 5-dag seedling had a total of six leaves, including the primordia (S3C Fig). In the 5 days after the 3-h DEX treatment, the DEX-treated seedlings initiated 1.05 ± 0.16 leaves on average, whereas mock-treated seedlings initiated 1.45 ± 0.11. The ratio of plants with each number of leaves at 10 dag is shown in Fig 2D. These data indicated that about 20% seedlings initiated no leaves after DEX treatment. Thus, leaf initiation also seemed to be affected by pulse downregulation of *OsWOX4*. Because the number of leaves varied between DEX- and mock-treated seedlings, we used seedlings with 7 leaves to compare histological characteristics in further analyses. (In the seedlings with 7 leaves at 10 dag, one plastochron was 5 days, and the 3^rd^, 4^th^ and 5^th^ leaves corresponded to P4, P3 and P2 primordia, respectively, in 5-dag seedlings before DEX treatment).

### *OsWOX4* is necessary for vascular development in leaves

Next, we examined the effect of pulse downregulation of *OsWOX4* on vascular differentiation. In wild-type leaf primordia, vascular differentiation initiates earlier in the central region than in the lateral region. To compare vascular bundles at the same developmental stage, we focused on only the large vascular bundle (LVB) in the center of P4 leaves (4^th^ leaf).

**Fig 3:**
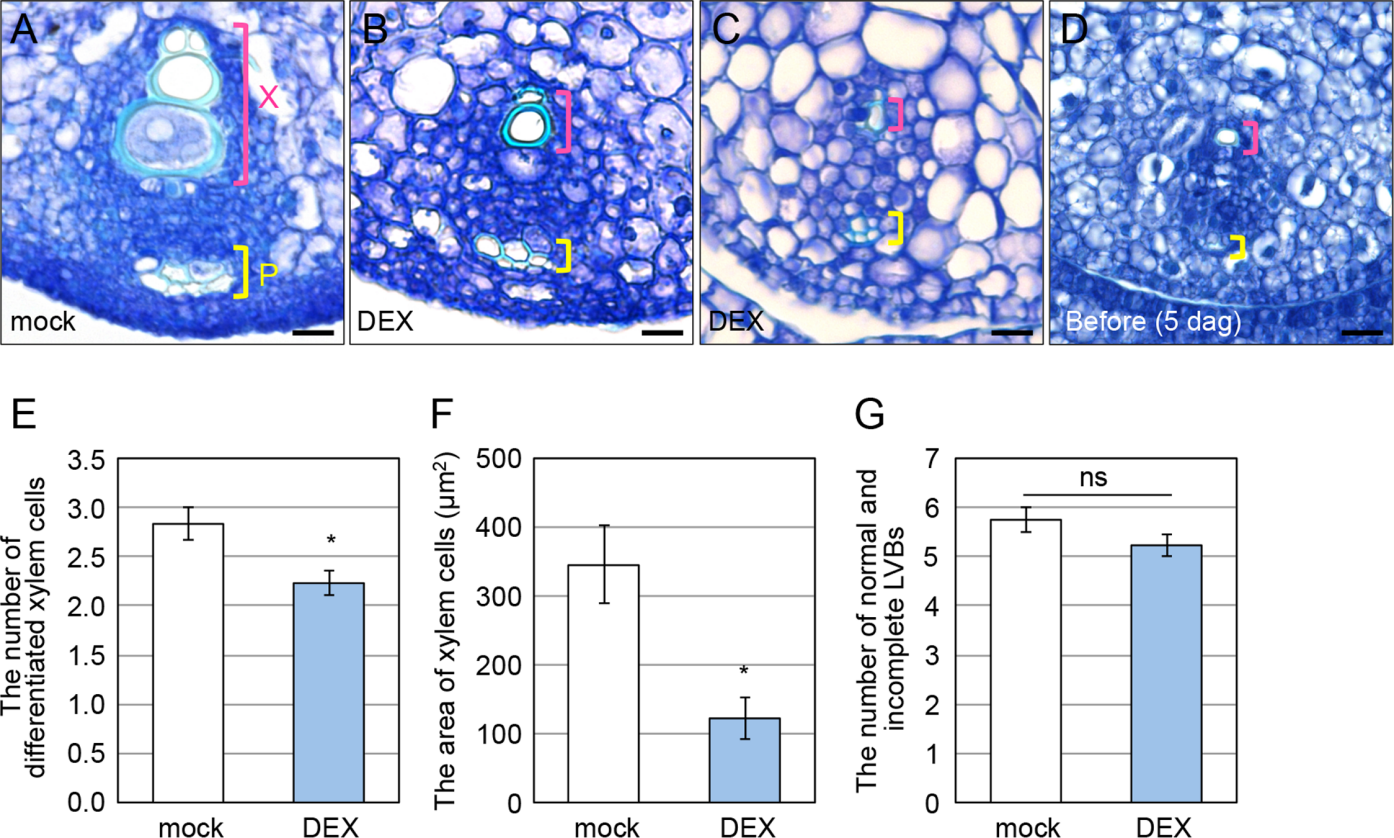
Effects of pulse downregulation of *OsWOX4* on vascular development in leaves. (A) to (D) Transverse sections of the central large vascular bundle (LVB) of a P4 leaf primordium in mock-treated (A) and DEX-treated (B and C) plants, and a P3 leaf primordium in a 5-dag plant before DEX treatment (D). The tissues were stained with toluidine blue. Bars = 10 μm. P, phloem; X, xylem. (E) Number of differentiated xylem cells in the central LVB of P4. (F) Area corresponding to xylem cells in the central LVB of P4. (G) Number of normal and incomplete LVBs in P4. Transgenic plants carrying *pACT1-GVG>OsWOX4*:*RNAi* were treated as indicated in Fig 2A. In (E) to (G), data are the mean ± SE (*n* = 12 [mock]; *n* = 13 [DEX]). Student’s t-test, *P < 0.01. ns, not significant.

In mock-treated plants, differentiated xylem and phloem were clearly observed in the central vascular bundle of P4 (Fig 3A). By contrast, undeveloped xylem and phloem were found in many of the DEX-treated plants (Fig 3B). In some severe cases, only a very small xylem cell was observed (Fig 3C). This histological phenotype was highly similar to that observed in 5-dag plants just before DEX treatment (Fig 3D), suggesting that vascular differentiation was almost completely inhibited by *OsWOX4* downregulation in this case. In some DEX-treated plants, cells were also less stained with toluidine blue, probably due to vacuolation (Fig 3C).

We performed a quantitative analysis on xylem cells, which are easily distinguished from other cells due to their thick cell walls. The number of xylem cells was significantly decreased by the downregulation of *OsWOX4* (Fig 3E). Furthermore, the area that the xylem cells occupied was markedly reduced (Fig 3F). These observations indicated that pulse downregulation of *OsWOX4* inhibited not only differentiation of the xylem but also its growth during vascular development. Thus, *OsWOX4* is likely to play an essential role in vascular differentiation in rice.

We also counted the number of normal LVBs and incomplete LVBs in P4 primordia, the latter of which contained aborted xylem and phloem. The total number of these LVBs did not differ significantly between mock- and DEX-treated plants (Fig 3G). At the time of DEX treatment, the P4 primordia were at the P3 stage. Because the onset of vascular differentiation continues to occur during the P3 to P4 stages under normal conditions [24], our observation suggests that vascular initiation is not affected by pulse downregulation of *OsWOX4*.

### *OsWOX4* downregulation affects vascular differentiation through cytokinin action

**Fig 4:**
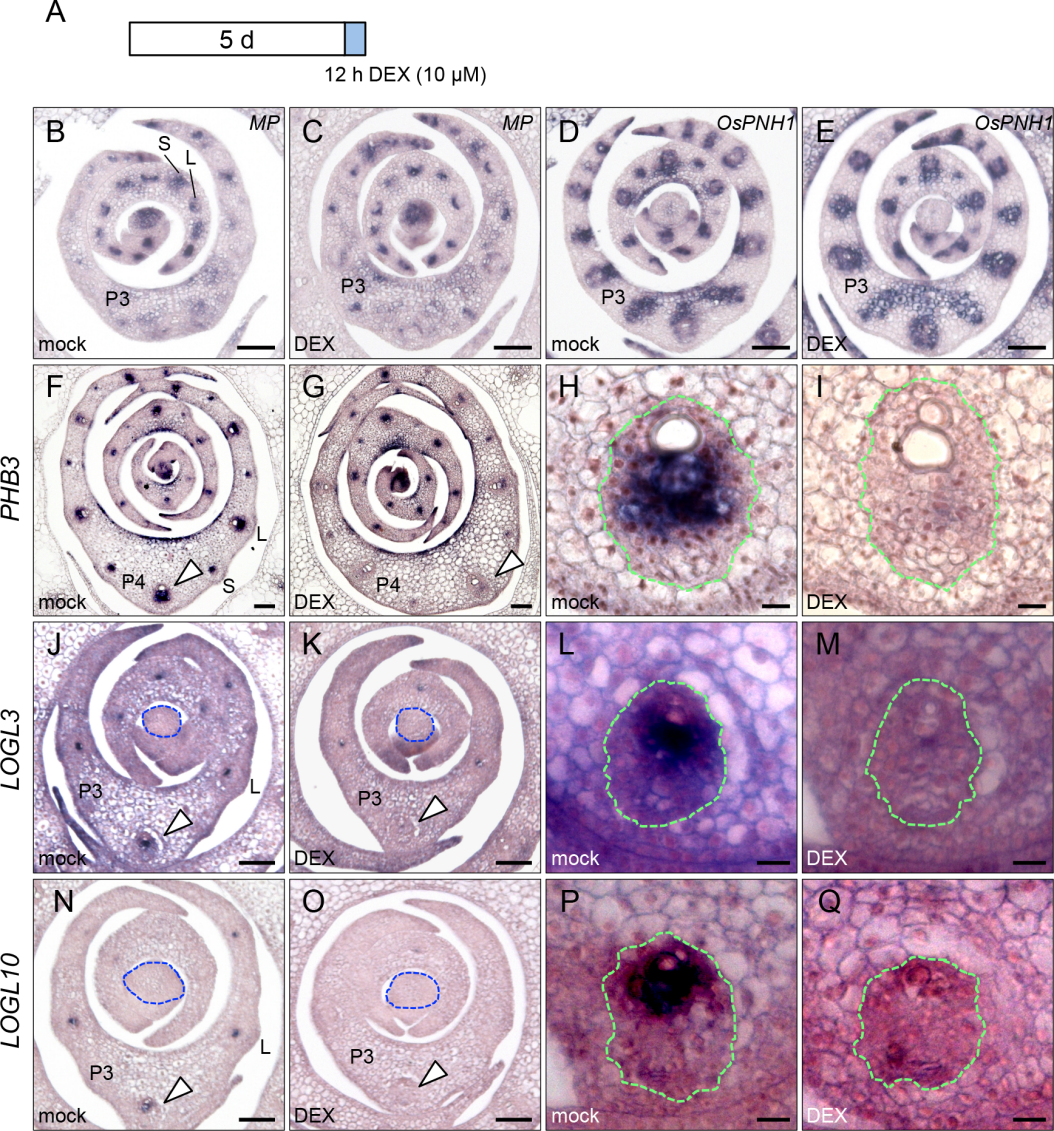
Effects of *OsWOX4* knockdown on the expression patterns of genes associated with vascular development in the shoot apex. (A) Schematic representation showing the DEX pulse treatment for in situ experiments. Transgenic plants (5 dag) carrying *pACT1-GVG>OsWOX4*:*RNAi* were treated with or without DEX for 12 h and the shoot apices were immediately fixed for subsequent in situ analysis. (B) and (C) In situ localization of *OsARF11/MP* transcript. (D) and (E) In situ localization of *OsPNH1* transcript. (F) to (I) In situ localization of *PHB3* transcript. (J) to (M) In situ localization of *LOGL3* transcript. (N) to (Q) In situ localization of *LOGL10* transcript. (H), (I), (L), (M), (P) and (Q) show a magnified view of the central vascular bundle indicated by the arrowhead in (F), (G), (J), (K), (N) and (O), respectively. Green dotted line indicates the vascular bundle in (H), (I), (L), (M), (P) and (Q). Blue dotted line indicates the SAM in (J), (K), (N) and (O). L, Large vascular bundle; S, small vascular bundle. Bars = 50 μm in (B) to (G), (J), (K), (N) and (O); 10 μm in (H), (I), (L), (M), (P) and (Q).

To elucidate how *OsWOX4* is involved in the genetic network regulating vascular development, we analyzed the effect of *OsWOX4* downregulation on the expression of several key genes associated with vascular development. In *Arabidopsis*, *MP* plays a key role in the initiation of vascular differentiation, whereas *PNH1* is reportedly expressed in vascular bundles including their future region [34, 36, 44]. Initially, therefore, we focused on the orthologs of these genes: *OsMP/OsARF11* and *OsPNH1* [24, 45]. For in situ experiments, 5-dag transgenic plants carrying *pACT1-GVG>OsWOX4*:*RNAi* were treated with DEX for 12 h, and then the shoot apices were immediately fixed for subsequent analysis (Fig 4A).

In mock-treated plants, *OsMP* was expressed in the future region of both LVBs and SVBs in P1 and P2 primordia, but this expression had largely disappeared from these bundles in the central region of P3 primordia (Fig 4B). This result suggests that *OsMP* is involved in early vascular development. *OsPNH1* was expressed in the developing vascular bundle of all primordia (Fig 4D), as previously reported [24]. Similar expression patterns of the two genes were observed in DEX-treated plants (Fig 4C and 4E), suggesting that downregulation of *OsWOX4* did not affect the expression of either gene.

Class III leucine zipper transcription factor (HD-ZIPIII) genes such as *PHABULOSA* (*PHB*) and *ARABIDOPSIS THALIANA HOMEOBOX8* are required for xylem cell differentiation in *Arabidopsis* [46, 47]. We therefore examined the expression patterns of rice *PHB*3 [48]. *PHB3* signals were clearly detected in both LVBs (P2-P4) and SVBs (P3-P4) in mock-treated plants (Fig 4F). By contrast, expression of *PHB3* was greatly reduced in P4 primordia of DEX-treated plants (Fig 4G); in particular, *PHB*3 signals in the central region completely disappeared after *OsWOX4* knockdown (Fig 4H and 4I). These results suggest that *OsWOX4* is required, in part, for *PHB3* expression.

In *Arabidopsis*, *LOG* genes such as *LOG3* and *LOG4* encoding the cytokinin-activating enzyme play a crucial role in vascular differentiation [39, 40, 42]. Thus, we examined the expression pattern of two rice *LOG* genes, *LOG-like3* (*LOGL3*) and *LOGL10*, which belong to the same clade as *Arabidopsis LOG3* and *LOG4* [42]. Both *LOGL3* and *LOGL10* were expressed in developing LVBs in mock-treated plants (Fig 4J and 4N). A close-up view indicated that their expression was localized to xylem precursor cells (Fig 4L and 4P). In DEX-treated plants, by contrast, *LOGL3* and *LOGL10* signals were decreased or had disappeared from several LVBs (Fig 4K, 4M, 4O and 4Q). These results suggest that *OsWOX4* promotes the expression of *LOGL3* and *LOGL10* in LVBs.

**Fig 5:**
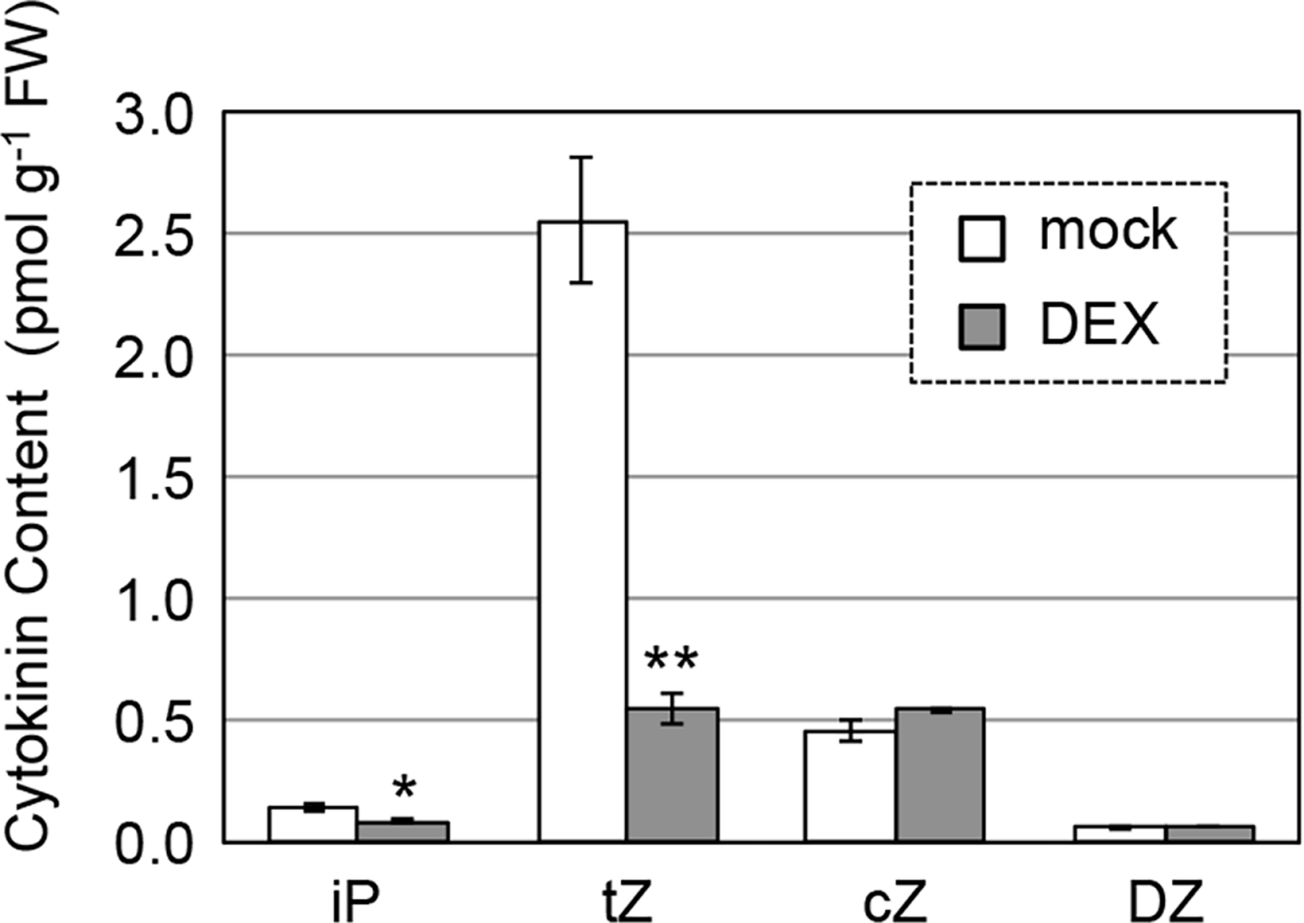
Effect of *OsWOX4* knockdown on cytokinin content. Transgenic plants (5 dag) carrying *pACT1-GVG>OsWOX4*:*RNAi* were treated with DEX for 12 h and then the amount of each type of cytokinin was measured as described in Materials and methods. iP, isopentenyladenine; tZ, *trans*-zeatin; cZ, *cis*-zeatin; DZ, dihydrozeatin. Data are the mean ± SE (*n* = 4 biological replicates). Student’s t-test, *P < 0.05, **P < 0.001.

Next, we examined the levels of several cytokinin forms such as isopentenyladenine (iP) and *trans*-Zeatin (tZ) in the shoot apex including P1–P3 primordia. The tZ content was markedly reduced in plants treated with DEX for 12 h as compared with mock-treated plants, and the iP content was also significantly decreased in DEX-treated plants (Fig 5). These results indicate that downregulation of *OsWOX4* leads to decreased cytokinin levels, probably due to reduced expression of rice *LOG* genes.

### *OsWOX4* is involved in cell proliferation required for midrib formation

**Fig 6:**
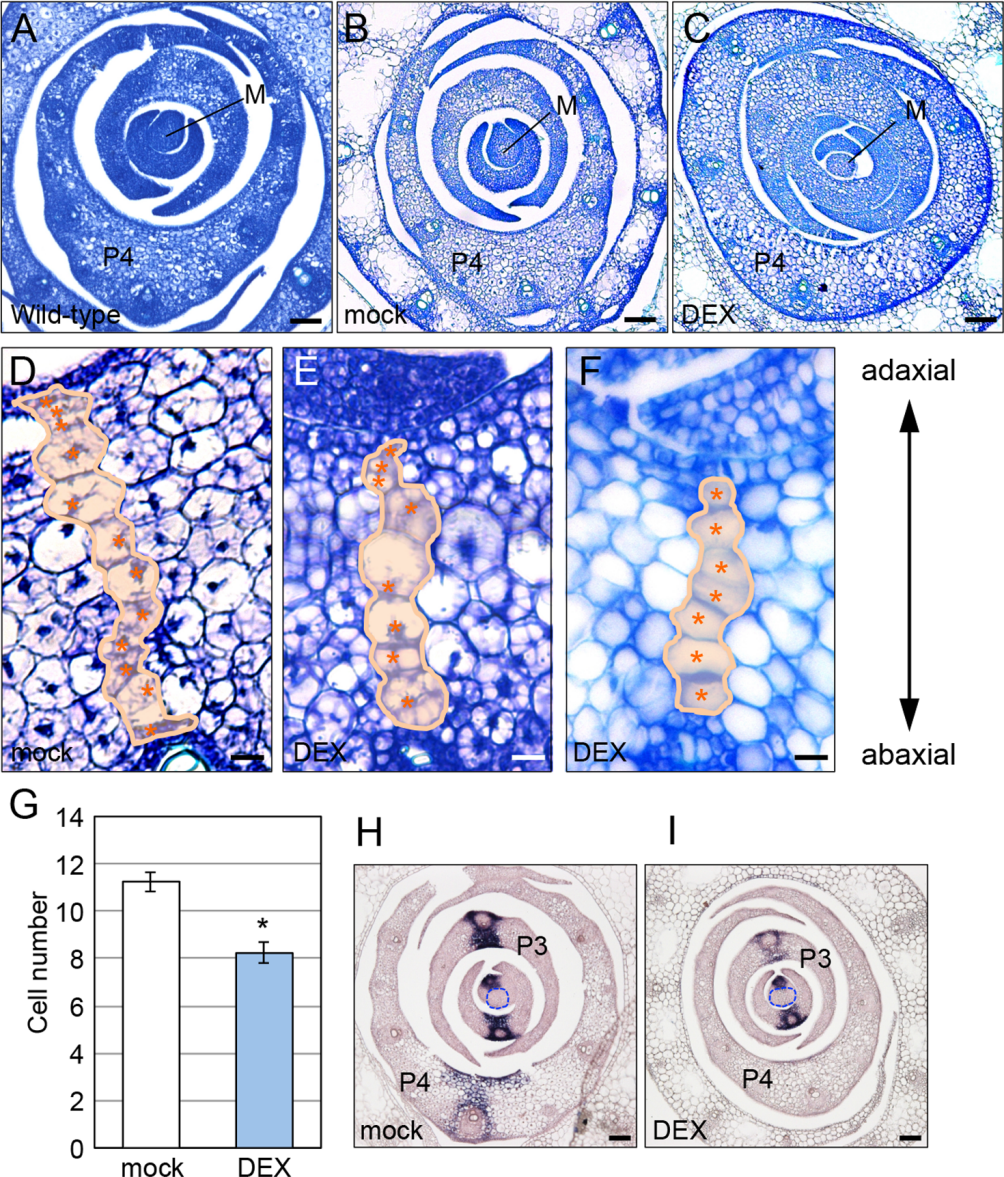
Effect of *OsWOX4* knockdown on the morphology of leaf primordia. (A) to (C) Transverse section of the shoot apex in a wild-type plant (A), and mock-treated (B) and DEX-treated (C) transgenic plants carrying *pACT1-GVG>OsWOX4*:*RNAi*. Tissues were stained with toluidine blue. M, SAM. (D) to (F) Central region of a P4 leaf primordium. Cells in the central file are indicated by false coloring and asterisks. (G) Number of cells in the central file of a P4 leaf primordium. Data are the mean ± SE (*n* = 12 [mock]; *n* = 13 [DEX]). Student’s t-test, *P < 10^−4^. (H) and (I) In situ localization of *DL* transcript. The SAM is indicated by the dotted line. Transgenic plants carrying *pACT1-GVG>OsWOX4*:*RNAi* were treated as indicated in Fig 2A (A to G) and in Fig 4A (H and I). Bars = 50 μm in (A) to (C), (H) and (I); 10 μm in (D) to (F).

We found that the morphology of leaf primordia was also affected by *OsWOX4* downregulation. The central region of the P3 and P4 primordia was thick, and the thickness was gradually reduced toward the lateral and marginal regions in both wild-type and mock-treated plants (Fig 6A and 6B). By contrast, the lateral regions of the P3 and P4 primordia became thicker in DEX-treated plants (Fig 6C). Quantitative analysis showed that the reduction in thickness in the lateral region was smaller in DEX-treated plants than in mock-treated plants (S4 Fig).

To examine the abnormality of leaf primordia in more detail, we counted the number of cells in a cell file of the central region of P4 primordia (Fig 6D to 6F). The cell number in this region was significantly lower in DEX-treated plants (Fig 6G), suggesting that pulse downregulation of *OsWOX4* partially inhibited cell proliferation in the central region of leaf primordia. The thickness of the central region was not significantly affected by DEX-treatment (S4B Fig), probably due to the abnormal cell expansion caused by *OsWOX4* downregulation.

Our previous work indicated that cell proliferation in the central region of leaf primordia is regulated by the *DL* gene and is associated with subsequent midrib formation [25, 26, 49]. We therefore examined the expression pattern of *DL* in *OsWOX4* knockdown plants. Consistent with previous reports, *DL* transcripts were detected in several cell files in the central region of leaf primordia (P1-P4) in mock-treated plants (Fig 6H). In DEX-treated plants (12 h), however, expression of *DL* in P3 primordia was markedly decreased; furthermore, no signal was detected in P4. By contrast, the reduction of *DL* expression in P1 and P2 primordia seemed to be small, especially in P1 (Fig 6I). These results suggest that *OsWOX4* is required to maintain *DL* expression in leaf primordia after the initiation of its expression.

### *OsWOX4* regulates the expression of a number of genes in leaf primordia

To elucidate further the effects of *OsWOX4* on gene expression, we performed transcriptome profiling. The *pACT1-GVG>OsWOX4*:*RNAi* transgenic plants were treated with DEX (or mock) for 3 or 12 h, and the shoot apices, including the SAM and leaf primordia, were harvested immediately. RNA isolation and microarray analysis were then carried out on three biological replicates.

**Fig 7:**
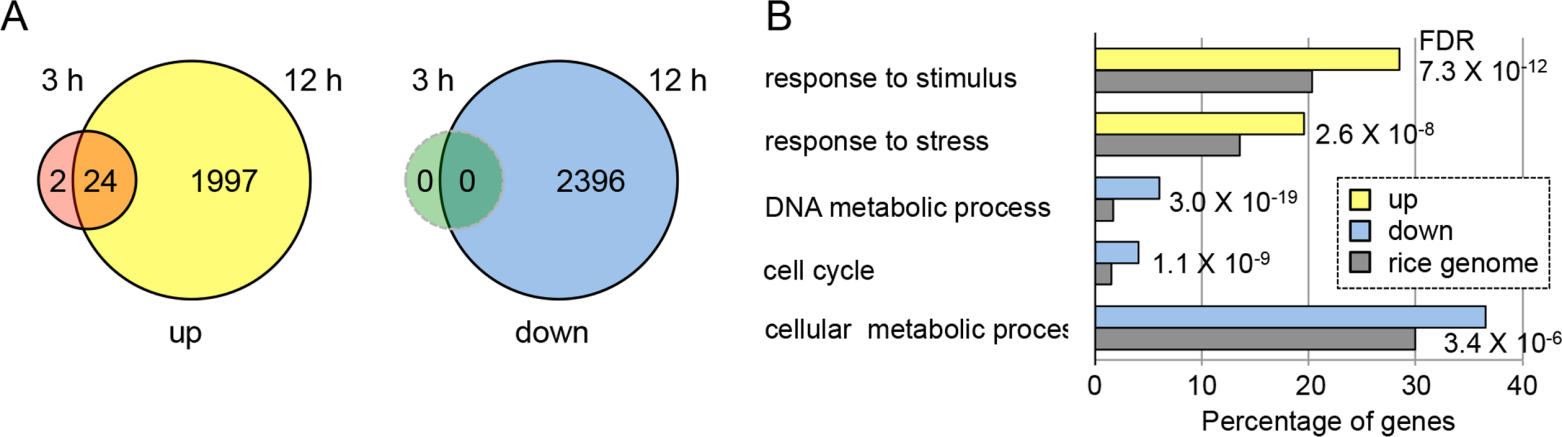
Transcriptome analysis to examine the effect of *OsWOX4* knockdown. (A) Number of upregulated and downregulated genes (fold change ≥ 2.0, P < 0.01). (B) Selected terms in gene ontology enrichment analysis of genes that were up- or downregulated genes after 12 h of DEX treatment. Transgenic plants carrying *pACT1-GVG>OsWOX4*:*RNAi* were treated with DEX for 3 or 12 h, and then microarray analysis was performed as described in Materials and methods.

In transgenic plants subjected to DEX treatment for 3 h, 26 genes were significantly upregulated and no genes were downregulated (fold change ≥ 2.0, P < 0.01) (Fig 7A). In those subjected to DEX treatment for 12 h, 2021 and 2396 genes were upregulated and downregulated, respectively (Fig 7A). Most genes that were upregulated after 3 h of DEX treatment were also upregulated after 12 h of treatment. Consistent with the results of *in situ* hybridization analysis, *LOGL3*, *LOGL10* and *DL* were found to be downregulated after 12 h of DEX treatment (S5 Fig). In addition, the microarray data indicated that downregulation by the *pACT1-GVG>OsWOX4*:*RNAi* construct was restricted to *OsWOX4*, and did not act on the other members of the *WOX* gene family (S1 Table).

To characterize the types of gene functioning downstream of *OsWOX4*, we performed a gene ontology (GO) enrichment analysis focusing on genes that were altered after 12 h of DEX treatment. Overall, 14 and 17 GO terms in the biological process category were significantly enriched among the upregulated and downregulated genes, respectively (S6 Fig). Notably, GO terms related to cell cycle, cellular metabolic process, and cellular component organization were highly enriched among the downregulated genes (Fig 7B and S6 Fig). In addition, terms related to metabolic processes associated with DNA and nucleic acid were also enriched among the downregulated genes. These results suggest that *OsWOX4* is required for cell activity and proliferation. On the other hand, terms related to response to stress and various stimuli were enriched among the upregulated genes (Fig 7B and S6 Fig).

We also noted that several genes related to jasmonic acid (JA) were included among the genes upregulated by *OsWOX4* silencing; for example, the genes encoding jasmonate ZIM-domain (JAZ) proteins, which negatively regulate JA signaling, and allene oxidase synthase (AOS) enzymes, which are central to JA biosynthesis, were upregulated (S7A and S7B Fig). In addition, an estimation of JA content showed that JA and JA-Ile were markedly increased in DEX-treated plants as compared with mock-treated plants (S7C and S7D Fig).

### *OsWOX4* is required for normal cell cycle regulation

**Fig 8:**
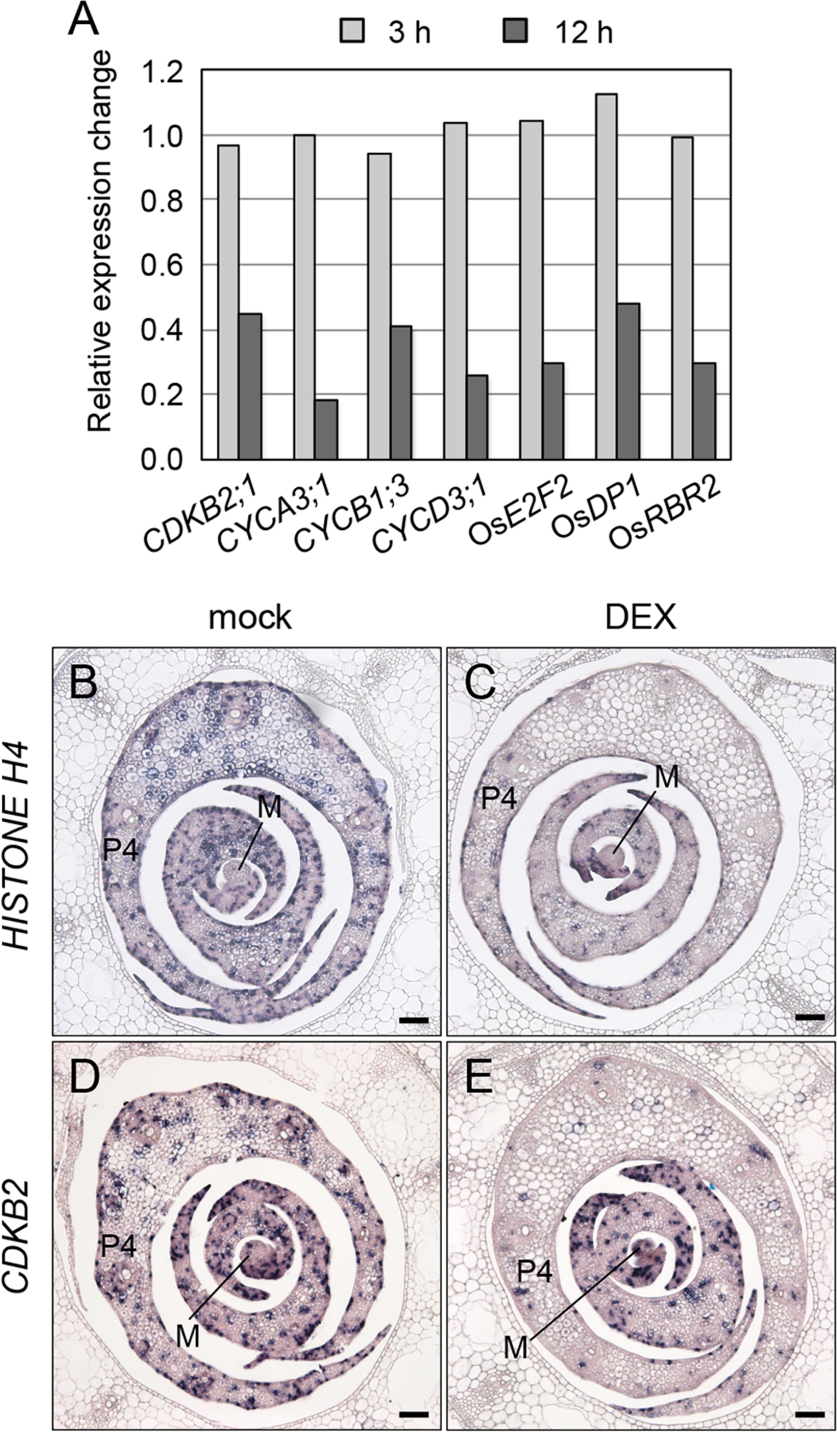
Effect of *OsWOX4* knockdown on the expression of cell cycle-related genes. (A) Expression levels of *CDKB2;1*, *CYCA3;1*, *CYCB1;3*, *CYCD3;1*, *OsE2F2*, *OsDP1* and *OsRBR2* relative to mock-treated samples in microarray analysis. (B) to (E) Expression pattern of *HISTONE H4* ([B] and [C]) and *CDKB2* ([D] and [E]) genes. Transgenic plants (5 dag) carrying *pACT1-GVG>OsWOX4*:*RNAi* were treated as indicated in Fig 4A. M, SAM. Bars = 50 μm.

On the basis of the GO enrichment analysis, we focused on genes involved in cell cycle regulation. Genes encoding B-type cyclin-dependent kinase (CDKB) and several cyclins that drive the cell cycle progression were markedly downregulated by 12 h of DEX treatment (Fig 8A). In addition, the expression of genes encoding core cell cycle regulators, including genes similar to *Arabidopsis E2F*, *E2F-DIMERIZATION PARTNER* (*DP*) and *RETINOBLASTOMA-RELATED* (*RBR*), was also reduced (Fig 8A) [50].

Next, we examined the spatial expression patterns of cell cycle-related genes, such as *HISTONE H4* as a maker of S-phase and *CDKB2* as a marker of G2/M phase [51, 52]. In mock-treated plants, both *HISTONE H4* and *CDKB2* were expressed in the leaf primordia from P1 to P4 (Fig 8B and 8D). After the knockdown of *OsWOX4* for 12 h, the expression of *HISTONE H4* in the leaf from P2 to P4 was markedly decreased; indeed, most *HISTONE H4* signals disappeared from these leaf primordia (Fig 8C). The expression of *CDKB2* was strongly decreased in P4, and moderately decreased in the inner leaf primordia (Fig 8E). Together, these results indicate that *OsWOX4* is required for normal cell cycle progression. Thus, *OsWOX4* might be involved in leaf growth largely through cell cycle regulation.

### Long-term *OsWOX4* knockdown leads to abnormally vacuolated cells

**Fig 9:**
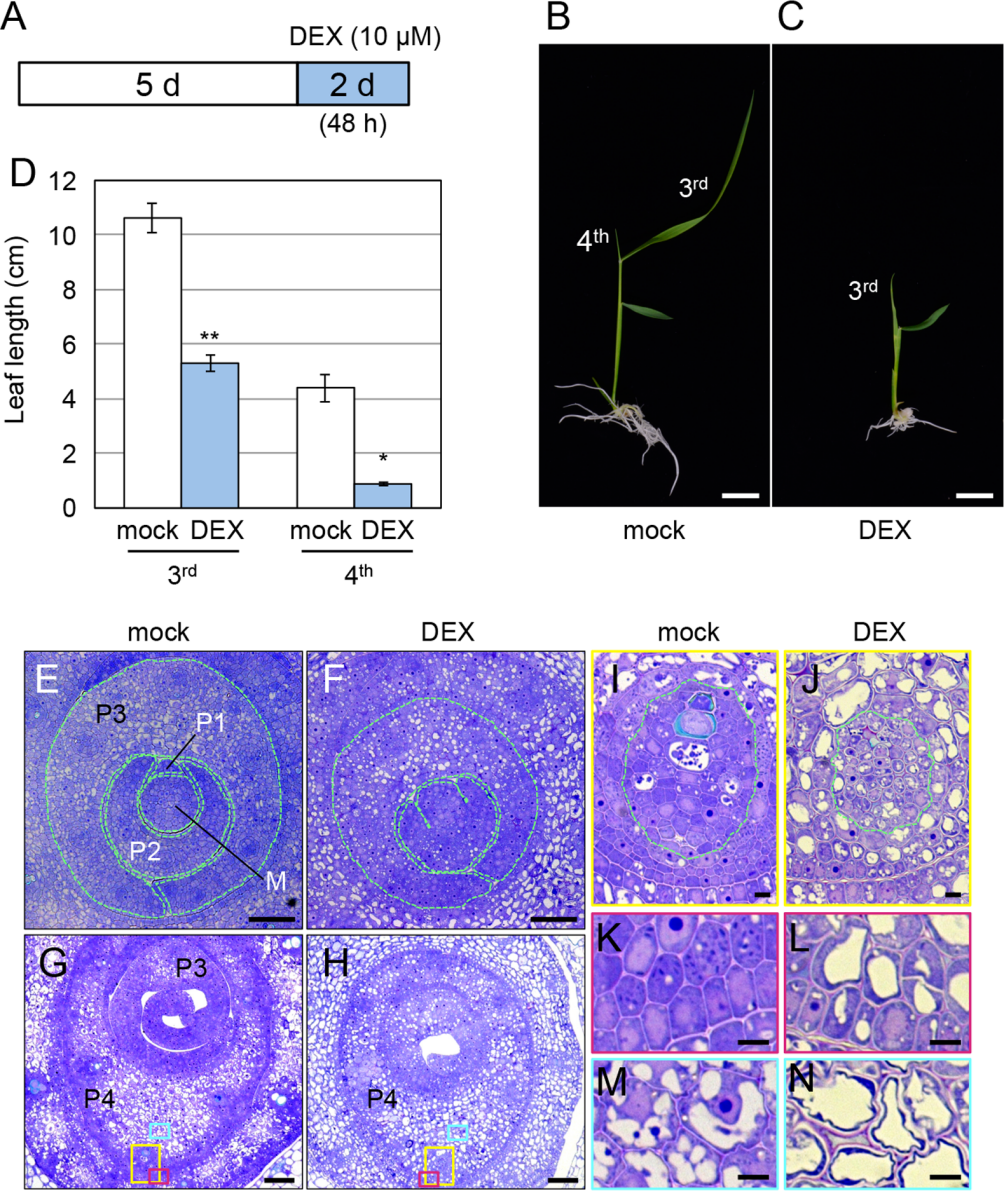
Effects of longer *OsWOX4* knockdown on cell activity. (A) Schematic representation showing the protocol for longer *OsWOX4* knockdown by DEX treatment. (B) and (C) Phenotype of seedlings after DEX or mock treatment. (D) Length of the 3^rd^ and 4^th^ leaves (total length of leaf blade and leaf sheath) of DEX- and mock-treated plants. Data are the mean ± SE (*n* = 6). Student’s t-test, *P < 10^−4^, **P < 10^−5^. (E) and (F) Shoot apex containing the SAM and leaf primordia (basal region). The SAM (M) and leaf primordia are outlined. (G) and (H) Shoot apex containing the leaf primordia above the SAM (apical region). (I), (K) and (M) Magnified views of the colored boxed regions shown in (G). (J), (L), and (N) Magnified views of the colored boxed regions shown in (H). The vascular bundle is outlined in (I) and (J). Transgenic plants carrying *pACT1-GVG>OsWOX4*:*RNAi* were treated as indicated in (A). Tissues were embedded in resin (Technovit 7100). Thin sections (0.7 μm) were generated and stained with toluidine blue. Bars = 1 cm in (B) and (C); 50 μm in (E) to (H); 5 μm in (I) to (N).

During histological analysis, we noticed that tissues from some plants treated with DEX for 3 h showed less staining with toluidine blue, although the frequency was low (4 out of 21; compare S8 Fig with Fig 6B). This observation suggested the possibility that cell activity was affected by *OsWOX4* knockdown. We therefore exposed plants to DEX for a longer period (48 h) and examined the effects (Fig 9).

Longer exposure to DEX caused more severe growth defects as compared with the 3-h pulse downregulation (Fig 9B to 9D). The shoot phenotype and the leaf length were almost indistinguishable between plants examined after treatment with DEX for 48 h and those examined before DEX treatment, suggesting that plant growth was almost completely inhibited by *OsWOX4* downregulation for 48 h (Fig 9C and 9D and S3A and S3B Fig). To observe the effect of *OsWOX4* knockdown at the cellular level, we prepared thin sections from resin-embedded shoot apices. In mock-treated plants, the P1 primordium was observed as a bulge from the SAM, and the P2 primordium was clearly distinguished from the SAM and P3 (Fig 9E). In DEX-treated plants, by contrast, the P1 primordium was not evident and the central region of the P2 primordium was fused to the SAM (Fig 9F and S9 Fig). This observation suggested that longer *OsWOX4* knockdown caused a serious defect in leaf primordium initiation.

In addition, white cells, which were not well stained with toluidine blue, were seen in both the basal and apical regions of P3 or subsequent leaf primordia in DEX-treated plants (Fig 9F and 9H). By contrast, such white cells were not evident in the basal region of these primordia in mock-treated plants (Fig 9E).

Close-up views showed that, in mock-treated plants, cells in the developing vascular bundles, and in epidermal and subepidermal layers were cytoplasmic-rich and vacuoles were inconspicuous (Fig 9I and 9K). In the inner tissues of P4, substantial amounts of cytoplasm remained, although some vacuoles were seen (Fig 9G and 9M). By contrast, large vacuoles were observed in many cells in all tissues in DEX-treated plants (Fig 9J, 9L and 9N). Furthermore, in the inner tissues, almost all cells contained an enlarged vacuole (Fig 9H and 9N). Together, these results indicated that longer *OsWOX4* knockdown strongly affected cellular activity in the leaf primordia.

## Discussion

### *OsWOX4* is a master regulator of leaf development in rice

We previously reported that *OsWOX4* is involved in SAM maintenance as a positive factor that promotes undifferentiated cell fate [20]. In this study, we focused on the function of *OsWOX4* in leaf development because *OsWOX4* is expressed in the leaf primordia in addition to the meristem. As a result, we revealed that *OsWOX4* has important functions in early leaf development in rice.

Because we analyzed leaf primordia that had differentiated from the SAM before *OsWOX4* knockdown was induced, it is unlikely that the phenotypes observed in the leaf primordia were a secondary effect caused by defects in SAM function. Rather, the phenotypes seem to result from the inhibition of leaf development itself.

Using our inducible *OsWOX4* knockdown system, we found that *OsWOX4* regulated the differentiation of leaf tissues related to both vascular development and midrib formation. In addition, transcriptome analysis showed that *OsWOX4* regulated the expression of a large set of downstream genes, including those related to cell cycle and cell division. Analysis of the spatial expression of these genes, coupled with detailed histological observation indicated that *OsWOX4* promoted cell activity and proliferation in early leaf development. Taking these findings together, we conclude that *OsWOX4* plays an important role in leaf development as a master regulator, which governs not only cell differentiation but also cell proliferation.

The transcriptome analysis also showed that 26 genes were upregulated by 3-h DEX treatment, but no genes were downregulated. This result suggests that *OsWOX4* probably acts as a transcriptional repressor, similar to *Arabidopsis* WOX proteins such as WUS, AtWOX5 and AtWOX7, although WUS acts as both a repressor and an activator of transcription [53–56]. However, it is also possible that some of the 26 genes are indirectly upregulated by *OsWOX4* knockdown. Further experiments such as ChiP seq and EMSA assay are required to examine whether early downregulated genes are direct or indirect targets of *OsWOX4*. A number of genes were up- or down-regulated by 12-h DEX treatment. Among these genes, we mainly focused on cell cycle-related or cytokinin-related genes, as described above and below. It is, however, unlikely that these genes were direct targets of *OsWOX4*, because their expression did not change significantly in a short time (3 h) after DEX application.

### Roles of *OsWOX4* in vascular development in rice leaf primordia

To examine the effect of *OsWOX4* downregulation on vascular development, we focused on the central LVB of P4, the cell fate of which should have already been determined for vascular differentiation at the P3 stage, when DEX treatment was applied. Our morphological analysis revealed that *OsWOX4* downregulation strongly inhibited vascular development in the leaf primordia. Differentiation of both the xylem and phloem was incomplete: for example, the number of the xylem cells was reduced and their growth was arrested in DEX-treated plants. By contrast, the number of LVBs including incomplete ones was unaffected by *OsWOX4* downregulation. Therefore, *OsWOX4* is likely to be involved in vascular development after its initiation.

Our understanding of the genes associated with vascular development in rice is limited. In this study, we examined the temporal and spatial expression patterns of putative genes responsible for vascular differentiation and then noted the effect of *OsWOX4* knockdown on these patterns during early leaf development in rice. In mock-treated plants, *OsMP* expression preceded the expression of *LOGL3* and *LOGL10*. In addition, *OsMP* was expressed in regions corresponding to future vascular bundles before they were histologically recognized (P1 and P2), whereas the two *LOGL* genes were expressed in putative xylem precursor cells in subsequent primordia stages. The difference in expression timing of the two genes is consistent with the fact that *LOG* genes are induced downstream of *MP* gene function in *Arabidopsis* [37, 39, 40], and suggests that the *MP* and *LOG* homologues function similarly in vascular development in both plants.

The expression of *LOGL3* and *LOGL10* was markedly reduced by *OsWOX4* knockdown. Consistent with this reduction, cytokinin levels were lower in *OsWOX4* knockdown than in mock-treated plants. Therefore, it is likely that *OsWOX4* is responsible for cytokinin synthesis through positive regulation of the *LOGL* genes. In contrast to the *LOGL* genes, the expression level of *OsMP* was not affected by *OsWOX4* downregulation. This result is consistent with the above inference that *OsWOX4* is unlikely to be involved in vascular initiation in rice. In *Arabidopsis*, *MP* is involved in the initial stage of vascular development by translating auxin signaling [33, 36, 57, 58]. Thus, rice *OsWOX4* seems to promote proliferation via positive regulation of the two *LOGL* genes once cells have been committed to a vascular fate by *OsMP*. The observed reduction of *PHB3* expression in *OsWOX4* knockdown plants further suggests the possibility that *OsWOX4* acts upstream of genes required for xylem formation.

In *Arabidopsis*, *AtWOX4* is required to maintain stem cells in the cambium in established vascular bundles [7, 12]. Unlike *Arabidopsis*, rice has no distinct cambium. *OsWOX4* seems to be involved at early stages of vascular differentiation through the promotion of cell proliferation via cytokinin biosynthesis. During rice evolution, therefore, it seems that the function of *OsWOX4* may have been recruited to contribute to vascular differentiation in another elaborate way.

### *OsWOX4* contributes to regulating *DL* expression, which is required for normal midrib formation

*OsWOX4* knockdown also affected the morphology of leaf primordia. In particular, the number of cells along the adaxial–abaxial axis in the central region of leaf primordia was significantly reduced by *OsWOX4* knockdown. Moreover, *DL* expression was downregulated in this region. *DL* is responsible for the proliferation of cells that form the midrib in the central region [25, 26, 49]. Therefore, it seems that *OsWOX4* promotes *DL* expression in order to acquire sufficient cells in this region for midrib formation.

In wild type, *DL* is expressed in several cell files in the central region of the P1 to P4 leaf primordia [25]. The downregulation of *DL* by *OsWOX4* knockdown seemed to depend on the leaf stage: the reduction in *DL* transcript was much higher in P3 and P4 than in P1 or P2. This result suggests that *OsWOX4* is mainly involved in maintenance rather than initial activation of *DL* expression. Multiple *cis* regulatory regions are reportedly required for proper expression of the *DL* gene [49]. For example, intron 2 is responsible for the early expression of *DL* in P1 and P2, whereas intron 1 is associated with quantitative regulation. The detailed molecular mechanism of *DL* expression still remains unknown. It will be interesting to determine how *OsWOX4* regulates *DL* expression in a manner dependent on the developmental stage of the leaf primordia.

Microarray analysis showed that the *AOS* genes, encoding key enzymes for JA biosynthesis, were upregulated by *OsWOX4* knockdown. This finding was consistent with the increased amounts of JA and JA-Ile measured in *OsWOX4* knockdown plants. Hibara et al. showed that JA content is associated with midrib formation [59]. In the *precocious* (*pre*) rice mutant, which has low JA content due to a defect in *AOS* gene, midrib formation is accelerated in earlier leaves; by contrast, treatment of wild-type rice with methyl jasmonate inhibits midrib formation. It is therefore possible that *OsWOX4* is involved in midrib formation by modulating JA biosynthesis.

### *OsWOX4* regulates cell cycle progression to promote leaf growth

Several cells were severely vacuolated in the leaf primordia after longer exposure to *OsWOX4* knockdown. Pulse downregulation of *OsWOX4* also caused similar effects, albeit to a lesser extent. These observations indicate that *OsWOX4* is required to maintain cellular activity in developing leaves, where cells are actively proliferating. Consistent with this, our microarray analysis revealed that *OsWOX4* affects the expression of many genes related to the cell cycle and cellular activity. In addition, spatial expression analysis showed that *HISTONE H4* and *CDKB2* transcripts were highly reduced in the leaf primordia after *OsWOX4* knockdown. These findings suggest that *OsWOX4* promotes cell proliferation through the regulation of cell cycle progression, leading to normal leaf development in rice.

In *Arabidopsis*, cytokinin is known to be involved in the regulation of cell cycle progression, and plays an important role in promoting cell proliferation in developing leaves [60–62]. As shown above, *OsWOX4* knockdown resulted in a reduction of cytokinin levels. Therefore, *OsWOX4* seems to control cell cycle progression in general parenchyma cells in leaf primordia, in addition to its role in vascular development, by raising cytokinin levels in rice.

### *OsWOX4* is essential for both leaf development and SAM maintenance

Our previous study indicated that the function of *OsWOX4* is partially associated with cytokinin action in the SAM, because constitutive expression of *OsWOX4* increases cytokinin levels and promotes shoot regeneration (including formation of the SAM) from calli in the absence of cytokinin [20]. In this study, we have shown that *OsWOX4* also plays important roles in leaf development, which are again associated with cytokinin action. Therefore, *OsWOX4* seems to regulate two distinct developmental processes by promoting cytokinin activity.

In *Arabidopsis*, *AtWOX4* function is restricted to maintaining vascular stem cells and *AtWOX4* is not expressed in the SAM [12]. Instead, SAM maintenance is regulated by *WUS* [16, 17]. The functions of the AtWOX4 and WUS proteins differ further in *Arabidopsis*: for example, *AtWOX4* does not rescue *wus* and *pressed flower1/wox3* mutations, whereas *WUS* can rescue both [16, 63–65]. In rice, *TAB1* (*WUS* ortholog) acts in the initial stages of axillary meristem development, but has no function in SAM maintenance [19]. Instead, rice *OsWOX4* regulates SAM maintenance [20]. Thus, members of the WOX gene family seem to have diversified in different ways in the evolutionary lineage of *Arabidopsis* and rice.

We have shown that *OsWOX4* acts as a key regulator in leaf development in addition to SAM maintenance [20]. By contrast, *Arabidopsis WOX4* functions in vascular development [12]. It will be interesting to determine the ancestral function of genes in the WOX4 clade and in particular the distribution of rice-type and *Arabidopsis*-type *WOX4* genes among angiosperms. Future studies on the molecular mechanisms underlying *WOX4* function in rice and other plants will also help to deepen our understanding of the function and diversification of *WOX* genes in plants.

## Materials and methods

### Plant materials and growth conditions

Transgenic lines carrying the *pACT1-GVG>OsWOX4*:*RNAi* construct have been described previously [20]. Taichung 65 was used as the host strain of the transgenic lines and as a wild-type control. Plants were grown in an NK system BIOTRON (LH-350S; Nippon Medical and Chemical Instruments) at 28°C.

### DEX treatment

For DEX treatment of plants from germination, sterilized seeds were germinated and grown for 5 days on filter papers immersed in liquid Murashige and Skoog (MS) medium containing DEX (10 μM) in petri dishes. For other DEX treatment, sterilized seeds were germinated and grown for 5 days under the same conditions but in the absence of DEX. The 5-dag seedlings were then cultured in 20 ml of MS medium with or without DEX (10 μM) in an Erlenmeyer flask with shaking at 70 rotations/minute (Double shaker BR-30; TAITEC). The period of the DEX treatment is indicated in the figures and figure legends. In the pulse downregulation experiments, seedlings were washed with water after DEX treatment (3 h) and further grown on soil for 5 days.

### Histological analysis

Tissues were fixed in 4% (w/v) paraformaldehyde and 0.25% (v/v) glutaraldehyde in 50 mM sodium phosphate buffer (pH 7.2) under vacuum and dehydrated in a graded ethanol series. For paraffin sections, samples were followed by ethanol/xylene series, and finally embedded in paraffin (Paraplast Plus; McCormick), and sectioned at 7 μm with a microtome (HM 335E; Microm). For resin sections, samples were embedded in Technovit 7100 (Heraeus Kulzer), and sectioned at a thickness of 0.7 μm with an ultramicrotome (Ultracut R; Leica). Sections were stained with Toluidine Blue O (Wako) and observed under a light microscope (BX50; Olympus).

### In situ hybridization

To generate probes for *OsMP*, *OsPNH1*, *LOGL3*, *LOGL10* and *CDKB2* transcripts, partial cDNA fragments were amplified by using the following primers. *OsMP*, 5’-CACCTGATGGAGGAAAGTCTGT-3’ and 5’-AGCTTCCACTCTGAACTGCCAG-3’; *OsPNH1*, 5’-AAGGTGAATCATTGGGCTTG-3’ and 5’-GCCAGTCTTGAGATGCAACA-3’; *LOGL3*, 5’-ACTTAAGCTAGCTCTGGGTGCTG-3’ and 5’-CCGGTTTATGATGGATGCCTA-3’; *LOGL10*, 5’-CATCGAAGCTGAACTGGGAGA-3’ and 5’-AGCCTCTCAACGCTTAGTTACACAC-3’; *CDKB2*, 5’-CCGGTTGACATCTGGTCTGT-3’ and 5’-AAGCACACTAAGCAGCATCCA-3’. The fragments were cloned into the pCRII-TOPO vector (Invitrogen). RNA was transcribed with T7 or SP6 RNA polymerase after linearization of the chimeric plasmid, and then labeled with digoxigenin using DIG RNA Labeling Mix (Roche). Probes for *OsWOX4* [20], *PHB3* [48], *DL* [25] and *HISTONE H4* [51] were prepared by using previously described plasmids. Tissue samples embedded in paraffin blocks were sectioned at 10 μm with a microtome (HM 335E; Microm). In situ hybridization, and immunological detection were performed by the methods described in Toriba et al. [66].

### Quantification of hormones

After DEX treatment of 5-dag plants for 12 h, shoot apices including the SAM and leaf primordia, which contained the whole of P1 and P2, most of P3, and the basal parts of P4 and P5, were harvested and used for the quantification, which was performed in biological quadruplicate. Extraction and determination of cytokinins and jasmonates were performed as described previously by using ultraperformance liquid chromatography–tandem mass spectrometry (AQITY UPLC system/Xevo-TQS; Waters) with an ODS column (Aquity UPLC BEH C18, 1.7 μm, 2.1 3 100 mm; Waters) [67, 68].

### Microarray experiments

After DEX treatment of 5-dag plants for 3 or 12 h, shoot apices were harvested (as above) and used for RNA isolation. Total RNA was extracted by using TRIsure (BIOLINE), treated with RNase-free DNase I (Takara), and purified by using the NucleoSpin RNA Plant Kit (Macherey-Nagel). Microarray analysis was performed in biological triplicate using the Rice (US) gene 1.0 ST array (Thermo Fisher Scientific). The sense-strand DNA target was prepared by using a WT Expression Kit (Thermo Fisher Scientific) and a GeneChip WT Terminal Labeling and Controls Kit (Thermo Fisher Scientific) in accordance with the manufacturers’ instructions. Hybridization, washing, and staining procedures were run on a Fluidics Station 450 (Thermo Fisher Scientific) with a GeneChip Hybridization, Wash, and Stain Kit (Thermo Fisher Scientific). GeneChips were scanned with a GeneChip Scanner 3000 7G (Thermo Fisher Scientific). Normalization was performed by using the standard settings for GeneChip Gene 1.0 ST arrays on Expression Console Version 1.3 (Thermo Fisher Scientific). The resulting data were analyzed via the Subio Platform (Subio). GO enrichment analysis was carried out by agriGO (http://bioinfo.cau.edu.cn/agriGO/index.php). GO terms with FDR < 0.05 were taken to be significantly enriched relative to the background of the rice genome (MSU7.0).

### Accession numbers

Sequence data from this article can be found in the GenBank/EMBL databases under the following accession numbers: JF836159 (*OsWOX4*), AK103452 (*OsMP*), AB081950 (*OsPNH1*), AK102183 (*PHB3*), AK099538 (*LOGL3*), AK108805 (*LOGL10*), AB106553 (*DL*), and AK059682 (*CDKB2*).

## Supporting information

S1 Table Expression levels of *WOX* gene members obtained by microarray analysis. (PDF)

S1 Fig Expression of *OsWOX4* mRNA in the inducible knockdown line. Transgenic plants (5 dag) carrying *pACT1-GVG>OsWOX4*:*RNAi* were treated with or without DEX for 12 h and mRNA levels of *OsWOX4* were analyzed by RT-PCR. UBQ was amplified as an internal control. The PCR amplification comprised 30 for *OsWOX4* and 25 for *UBQ*.
(PDF)

S2 Fig Effect of DEX treatment on wild type. (A) and (B) Wild-type plants were treated with DEX for 5 days from germination. Bars = 1 cm.
(PDF)

S3 Fig Phenotype of plants before DEX induction. (A) Phenotype of the seedling at 5 dag. Bar = 1 cm. (B) Length of the 3^rd^ and 4^th^ leaves of seedlings at 5 dag. Data are the mean ± SE (*n* = 6). (C) Transverse section of the shoot apex of seedlings at 5 dag. The section was stained with toluidine blue. Bar = 50 μm. Transgenic plants carrying *pACT1-GVG>OsWOX4*:*RNAi* at 5 dag (before DEX treatment) were examined.
(PDF)

S4 Fig Quantification of abnormal leaf primordia caused by *OsWOX4* knockdown. (A) Diagram illustrating a transverse section of the leaf primordium. The thickness of the central (a) and lateral (b) regions is indicated by the dashed line. Light gray ellipses indicate LVB. (B) The thickness of the central region of P4 (“a” in S4A Fig). (C) Thickness of the lateral regions of P4 (“b” in S4A Fig). (D) Ratio of the thickness (lateral (b)/central (a)). In (B) to (D), data are the mean ± SE (*n* = 12 [mock]; *n* = 13 [DEX]). Student’s t-test, *P < 0.05, **P < 10^−3^. ns, not significant.
(PDF)

S5 Fig Relative change in expression levels of *LOGL3*, *LOGL10* and *DL* caused by *OsWOX4* knockdown. Expression levels were measured relative to mock-treated samples by microarray analysis.
(PDF)

S6 Fig Gene ontology (GO) categories enriched by *OsWOX4* knockdown. Significantly enriched GO terms (FDR < 0.05) were obtained on the basis of the microarray analysis, in which 2021 and 2396 genes were up- and downregulated, respectively, after *OsWOX4* knockdown for 12 h.
(PDF)

S7 Fig Effect of *OsWOX4* knockdown on the expression of JA-related genes and JA content. (A) and (B) Expression levels of *OsJAZ* genes (A) and *OsAOS* genes (B) relative to mock-treated samples in microarray analysis. (C) and (D) Amount of JA (C) and JA-Ile (D). Transgenic plants (5 dag) carrying *pACT1-GVG>OsWOX4*:*RNAi* were treated with DEX for 12 h and then analyzed. JA, jasmonic acid; JA-Ile, jasmonoyl-l-isoleucine. Data are the mean ± SE (*n* = 4 biological replicates). Student’s t-test, *P < 0.01, **P < 0.001.
(PDF)

S8 Fig A severe example of the effect on cell activity caused by pulse downregulation of *OsWOX4*. Transgenic plants carrying *pACT1-GVG>OsWOX4*:*RNAi* were treated as indicated in Fig 2A. Shown is a transverse section of a plant showing the phenotype of reduced staining with toluidine blue. Bar = 50 μm.
(PDF)

S9 Fig Effects of *OsWOX4* knockdown on the emergence of leaf primordia from the SAM. (A) Magnified view of the SAM and P1 and P2 leaf primordia shown in Fig 9E. (B) Magnified view of the SAM and leaf primordia shown in Fig 9F. Transgenic plants carrying *pACT1-GVG>OsWOX4*:*RNAi* were treated as indicated in Fig 9A. Tissues were embedded in resin (Technovit 7100). Thin sections (0.7 μm) were generated and stained with toluidine blue. Bars = 5 μm.
(PDF)

S1 Dataset Numerical data for graphs. (PDF)
