# Supplementary material for: WUSCHEL-RELATED HOMEOBOX4 acts as a key regulator in early leaf development in rice
Source: PLoS Genet. 2018 Apr 23;14(4):e1007365. doi: 10.1371/journal.pgen.1007365 (PMC5933814; doi:10.1371/journal.pgen.1007365)
Supplement: S1 Table — (PDF) [file pgen.1007365.s001.pdf]

S1 Table. Expression levels of *WOX* gene members obtained by microarray analysis

	Average ± SE			
	3 h		12 h	
	-DEX	+DEX	-DEX	+DEX
TAB1 (*OsWUS*)	41 ± 1.5	39 ± 0.6	46 ± 3.0	52 ± 2.7
WOX2	39 ± 2.5	41 ± 1.9	42 ± 2.3	60 ± 1.5
OsWOX3	167 ± 2.7	166 ± 1.4	179 ± 5.1	144 ± 3.8
QHB (*OsWOX5*)	17 ± 0.5	20 ± 2.1	18 ± 2.2	269 ± 36.5
OsWOX8/9	23 ± 3.3	26 ± 1.8	28 ± 2.1	41 ± 7.0
	17 ± 0.6	18 ± 0.5	15 ± 0.8	19 ± 0.9
OsWOX11/12	14 ± 1.3	16 ± 1.0	14 ± 0.9	16 ± 1.8
	44 ± 3.1	38 ± 0.7	36 ± 1.7	34 ± 0.6
	153 ± 21.4	109 ± 16.6	164 ± 19.3	117± 12.4
OsWOX13	175 ± 7.2	206 ± 13.1	190 ± 12.7	158 ± 7.2
