# Supplementary figures and images for: WUSCHEL-RELATED HOMEOBOX4 acts as a key regulator in early leaf development in rice
Source: PLoS Genet. 2018 Apr 23;14(4):e1007365. doi: 10.1371/journal.pgen.1007365 (PMC5933814; doi:10.1371/journal.pgen.1007365)

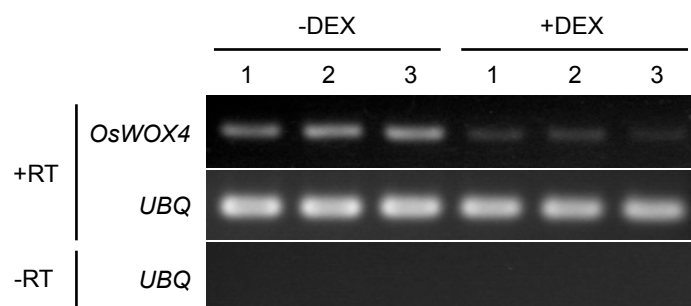

Supplement: S1 Fig — Transgenic plants (5 dag) carrying pACT1-GVG>OsWOX4:RNAi were treated with or without DEX for 12 h and mRNA levels of OsWOX4 were analyzed by RT-PCR. UBQ was amplified as an internal control. The PCR amplification comprised 30 for OsWOX4 and 25 for UBQ. (PDF) [file pgen.1007365.s002.pdf]

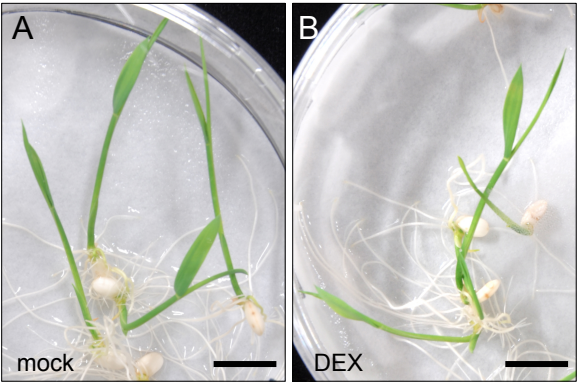

Supplement: S2 Fig — (A) and (B) Wild-type plants were treated with DEX for 5 days from germination. Bars = 1 cm. (PDF) [file pgen.1007365.s003.pdf]

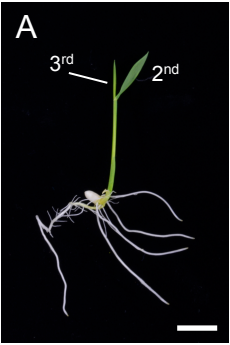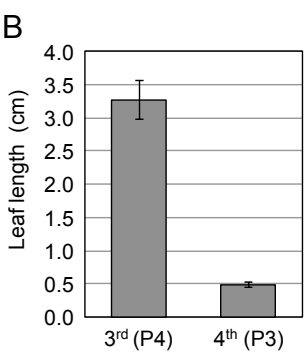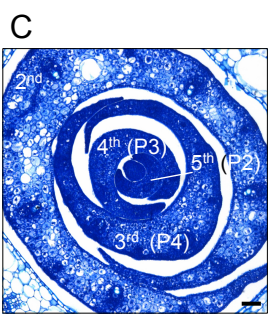

Supplement: S3 Fig — (A) Phenotype of the seedling at 5 dag. Bar = 1 cm. (B) Length of the 3rd and 4th leaves of seedlings at 5 dag. Data are the mean ± SE (n = 6). (C) Transverse section of the shoot apex of seedlings at 5 dag. The section was stained with toluidine blue. Bar = 50 μm. Transgenic plants carrying pACT1-GVG>OsWOX4:RNAi at 5 dag (before DEX treatment) were examined. (PDF) [file pgen.1007365.s004.pdf]

A

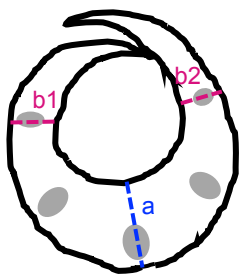

B

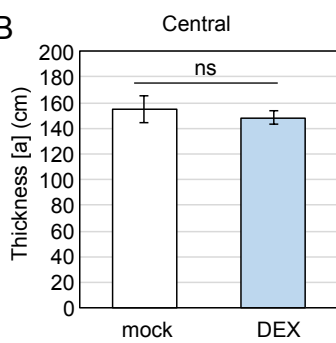

C

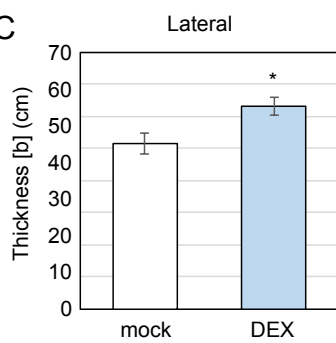

D

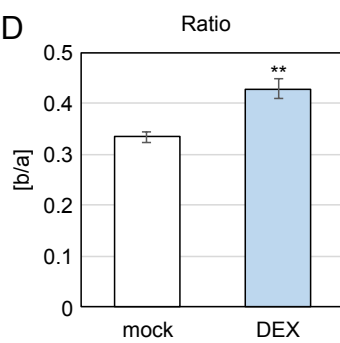

Supplement: S4 Fig — (A) Diagram illustrating a transverse section of the leaf primordium. The thickness of the central (a) and lateral (b) regions is indicated by the dashed line. Light gray ellipses indicate LVB. (B) The thickness of the central region of P4 (“a” in S4A Fig). (C) Thickness of the lateral regions of P4 (“b” in S4A Fig). (D) Ratio of the thickness (lateral (b)/central (a)). In (B) to (D), data are the mean ± SE (n = 12 [mock]; n = 13 [DEX]). Student’s t-test, *P < 0.05, **P < 10−3. ns, not significant. (PDF) [file pgen.1007365.s005.pdf]

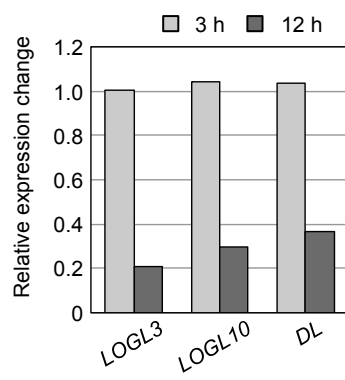

Supplement: S5 Fig — Expression levels were measured relative to mock-treated samples by microarray analysis. (PDF) [file pgen.1007365.s006.pdf]

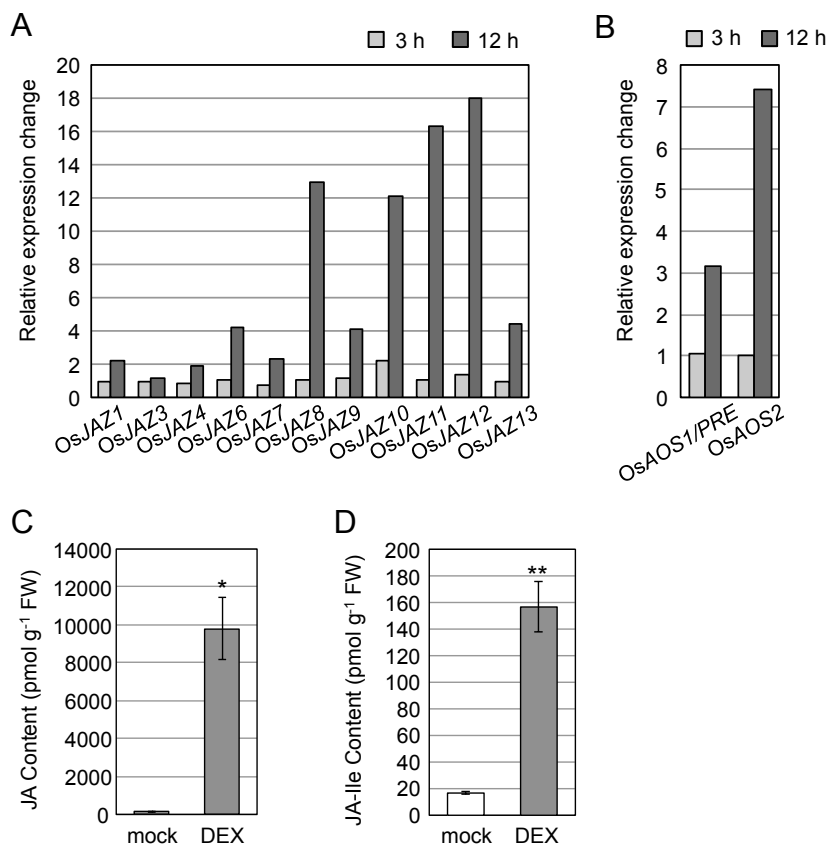

Supplement: S7 Fig — (A) and (B) Expression levels of OsJAZ genes (A) and OsAOS genes (B) relative to mock-treated samples in microarray analysis. (C) and (D) Amount of JA (C) and JA-Ile (D). Transgenic plants (5 dag) carrying pACT1-GVG>OsWOX4:RNAi were treated with DEX for 12 h and then analyzed. JA, jasmonic acid; JA-Ile, jasmonoyl-l-isoleucine. Data are the mean ± SE (n = 4 biological replicates). Student’s t-test, *P < 0.01, **P < 0.001. (PDF) [file pgen.1007365.s008.pdf]

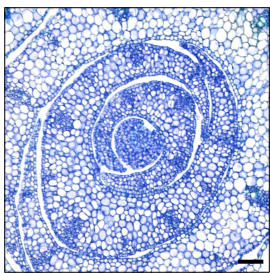

Supplement: S8 Fig — Transgenic plants carrying pACT1-GVG>OsWOX4:RNAi were treated as indicated in Fig 2A. Shown is a transverse section of a plant showing the phenotype of reduced staining with toluidine blue. Bar = 50 μm. (PDF) [file pgen.1007365.s009.pdf]

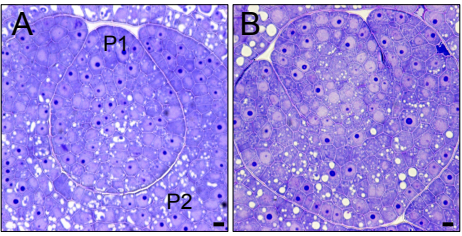

Supplement: S9 Fig — (A) Magnified view of the SAM and P1 and P2 leaf primordia shown in Fig 9E. (B) Magnified view of the SAM and leaf primordia shown in Fig 9F. Transgenic plants carrying pACT1-GVG>OsWOX4:RNAi were treated as indicated in Fig 9A. Tissues were embedded in resin (Technovit 7100). Thin sections (0.7 μm) were generated and stained with toluidine blue. Bars = 5 μm. (PDF) [file pgen.1007365.s010.pdf]
