# Supplementary material for: WUSCHEL-RELATED HOMEOBOX4 acts as a key regulator in early leaf development in rice
Source: PLoS Genet. 2018 Apr 23;14(4):e1007365. doi: 10.1371/journal.pgen.1007365 (PMC5933814; doi:10.1371/journal.pgen.1007365)
Supplement: S6 Fig — Significantly enriched GO terms (FDR < 0.05) were obtained on the basis of the microarray analysis, in which 2021 and 2396 genes were up- and downregulated, respectively, after OsWOX4 knockdown for 12 h. (PDF) [file pgen.1007365.s007.pdf]

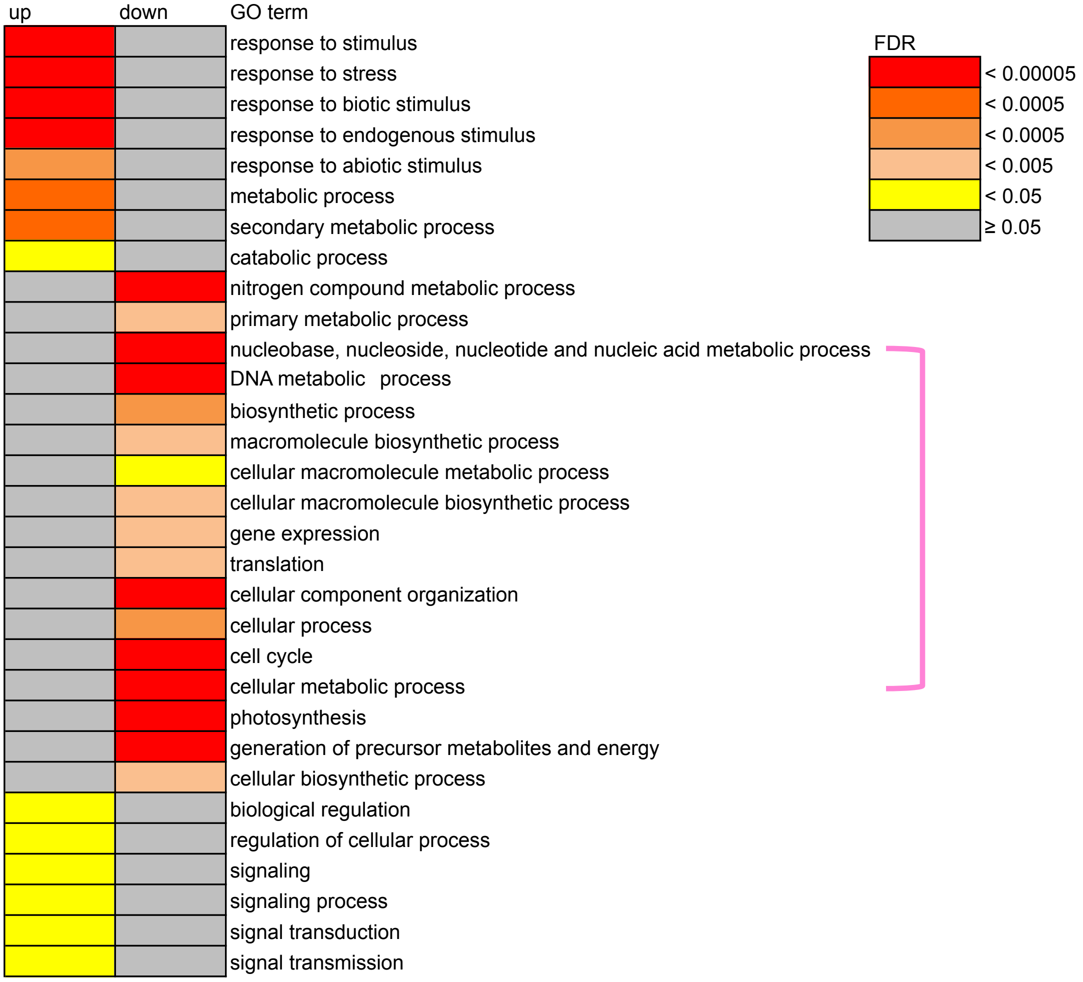
up
down
GO term
response to stimulus
response to stress
response to biotic stimulus
response to endogenous stimulus
response to abiotic stimulus
metabolic process
secondary metabolic process
catabolic process
nitrogen compound metabolic process
primary metabolic process
nucleobase, nucleoside, nucleotide and nucleic acid metabolic process
DNA metabolic process
biosynthetic process
macromolecule biosynthetic process
cellular macromolecule metabolic process
cellular macromolecule biosynthetic process
gene expression
translation
cellular component organization
cellular process
cell cycle
cellular metabolic process
photosynthesis
generation of precursor metabolites and energy
cellular biosynthetic process
biological regulation
regulation of cellular process
signaling
signaling process
signal transduction
signal transmission
FDR
< 0.00005
< 0.0005
< 0.0005
< 0.005
< 0.05
≥ 0.05
