# Supplementary material for: WUSCHEL-RELATED HOMEOBOX4 acts as a key regulator in early leaf development in rice
Source: PLoS Genet. 2018 Apr 23;14(4):e1007365. doi: 10.1371/journal.pgen.1007365 (PMC5933814; doi:10.1371/journal.pgen.1007365)
Supplement: S1 Dataset — (PDF) [file pgen.1007365.s011.pdf]

Data for Fig 2C

The length of the 3rd, 4th and 5th leaves of DEX- and mock-treated plants

	3rd leaf		4th leaf		5th leaf	
	-DEX	+DEX	-DEX	+DEX	-DEX	+DEX
average	13.09	6.62	11.74	7.44	6.22	1.50
SE	0.25	0.30	0.92	0.47	0.52	0.44
n=	16	17	16	17	9	10
	13.6	5.2	12.4	6.5	6.3	2.2
	12.3	5.1	15.4	5.4	5.3	0.2
	13.4	5.1	6.4	5.8	7.1	4.1
	14.2	5.8	6.5	7.3	3.6	0.3
	13.0	4.4	12.8	3.8	6.1	0.4
	12.9	5.5	5.6	4.7	7.2	2.1
	12.6	7.7	8.4	7.6	5.6	1.6
	14.0	8.4	15.0	8.5	9.2	3.3
	13.3	6.7	14.4	8.0	5.6	0.5
	14.6	7.1	16.0	10.5		0.3
	12.7	8.0	14.5	10.6		
	12.2	7.0	15.4	6.8		
	14.2	6.8	13.7	8.8		
	13.3	7.7	13.2	8.3		
	12.6	7.8	7.2	8.6		
	10.5	7.9	10.9	9.4		
		6.3		5.9		

Data for Fig 3E
The number of differentiated xylem cells in the central LVB of P4

	-DEX	+DEX
average	2.8	2.2
SE	0.17	0.12
n=	12	13
	3	2
	4	2
	3	2
	2	3
	3	2
	3	2
	3	2
	3	2
	3	3
	2	2
	3	2
	2	2
		3

Data for Fig 3F
The area of xylem cells in the central LVB of P4

	-DEX	+DEX
average	345.0	122.2
SE	56.6	30.7
n=	12	13
	434.3	13.0
	491.8	75.7
	615.8	57.0
	329.4	90.1
	491.9	26.4
	385.3	18.2
	628.6	114.9
	174.6	37.2
	115.0	149.5
	162.1	129.1
	276.2	367.5
	34.8	298.9
		211.5

Data for Fig 3G

The number of normal and incomplete LVBs in P4

	-DEX	+DEX
average	5.8	5.2
SE	0.25	0.23
n=	12	13
	5	5
	6	5
	7	5
	6	5
	5	4
	5	5
	5	5
	5	5
	5	5
	6	5
	7	7
	7	7
		5

Data for Fig 5
Cytokinin content in DEX- and mock-treated plants

	iP		tZ		cZ		DZ	
	-DEX	+DEX	-DEX	+DEX	-DEX	+DEX	-DEX	+DEX
average	0.142	0.089	2.554	0.546	0.459	0.543	0.061	0.067
SE	0.015	0.004	0.257	0.060	0.042	0.012	0.005	0.004
n=	4	4	4	4	4	4	4	4
	0.186	0.094	2.462	0.520	0.434	0.520	0.072	0.074
	0.118	0.097	1.867	0.721	0.373	0.524	0.062	0.066
	0.133	0.088	3.014	0.494	0.452	0.565	0.050	0.071
	0.130	0.078	2.873	0.449	0.575	0.561	0.057	0.056

Data for Fig 6G

The number of cells in the central file of the P4 leaf primordium

	-DEX	+DEX
average	11.3	8.2
SE	0.43	0.46
n=	12	13
	11	8
	13	8
	12	8
	10	8
	12	7
	12	12
	13	7
	12	8
	10	7
	12	6
	8	11
	10	9
		8

Data for Fig 9D
The length of the 3rd and 4th leaves of DEX- and mock-treated plants

	3rd leaf		4th leaf	
	-DEX	+DEX	-DEX	+DEX
average	10.62	5.28	4.40	0.88
SE	0.54	0.30	0.51	0.08
n=	6	6	6	6
	11.0	4.7	4.5	0.6
	12.8	6.0	6.2	1.0
	9.9	5.5	4.7	0.9
	9.2	5.0	4.2	0.8
	11.2	6.2	4.5	1.2
	9.6	4.3	2.3	0.8
